# Annual soil CO_2_ efflux in a cold temperate forest in northeastern China: effects of winter snowpack and artificial nitrogen deposition

**DOI:** 10.1038/srep18957

**Published:** 2016-01-06

**Authors:** Boqi Liu, Changcheng Mou, Guoyong Yan, Lijian Xu, Siling Jiang, Yajuan Xing, Shijie Han, Jinghua Yu, Qinggui Wang

**Affiliations:** 1 Center for Ecological Research, Northeast Forestry University, 26 Hexing Road, Harbin, 150040, China; 2 College of Agricultural Resource and Environment, Heilongjiang University, 74 Xuefu Road, Harbin, 150080, China; 3 Institute of forestry science of Heilongjiang province, 134 Haping Road, Harbin 150081, China; 4 State key laboratory of forest and ecology, Chinese Academy of Sciences, 72 Wenhua Road, Shenyang, 110015, China

## Abstract

We conducted a snow depth 0 cm (non-snowpack), 10 cm, 20 cm, 30 cm and natural depth) gradient experiment under four quantities of nitrogen addition (control, no added N; low-N, 5 g N m^−2^ yr^−1^; medium-N, 10 g N m^−2^ yr^−1^; and high-N, 15 g N m^−2^ yr^−1^) and took an-entire-year measurements of soil respiration (*R*s) in Korean pine forests in northeastern China during 2013–2014. No evidence for effects of N on *R*s could be found during the growing season. On the other hand, reduction of snowpack decreased winter soil respiration due to accompanied relatively lower soil temperature. We found that winter temperature sensitivities (*Q*_10_) of *R*s were significantly higher than the growing season *Q*_10_ under all the N addition treatments. Moderate quantities of N addition (low-N and medium-N) significantly increased temperature sensitivities (*Q*_10_) of *R*s, but excessive (high-N) addition decreased it during winter. The Gamma empirical model predicted that winter *R*s under the four N addition treatments contributed 4.8. ± 0.3% (control), 3.6 ± 0.6% (low-N), 4.3 ± 0.4% (medium-N) and 6.4 ± 0.5% (high-N) to the whole year *R*s. Our results demonstrate that N deposition will alter *Q*_10_ of winter *R*s. Moreover, winter *R*s may contribute very few to annual *R*s budget.

Soil respiration (*R*s) is the second largest carbon (C) flux (68–80 Pg C yr^−1^) between the atmosphere and terrestrial ecosystem^1^. Unprecedented global warming and nitrogen (N) deposition can impact *R*s through the change in soil temperature^2^ and nutrient availability^3^. How this second largest C flux responds to the changes of soil temperature and nutrient availability will exert substantial influence on global C cycle and climate feedbacks^4^,^5^. Even though many previous studies have explored the main effects of warming^1^,^6^ and nutrient availability^5^,^7^,^8^, a multifactor manipulative experiment is still needed to develop a comprehensive understanding of *R*s dynamics under the changes in these driving factors.

N deposition has been considered as a potential driving factor caused by global change, which exerts various effects on forest ecosystem processes in the cold temperate forests in northeastern China^9^. Many previous studies have reported that additions of N into forest soils exerted various effects (promoted, inhibited and unchanged) on *R*s rates and its temperature sensitivity^10^,^11^,^12^,^13^. Understanding the season patterns of temperature sensitivity of soil respiration and its effect factors is very important to quantify the C cycle. In cold temperate forests and boreal forests, previous field experiments of the N deposition effect on CO_2_ flux have been performed during the growing season^14^,^15^, and it is partly responded the *R*s of whole year. However, snowpack exerts pronounced impacts on soil respiration in winter^16^, and also the changes of increasing N deposition to *R*s during both winter and the growing season in these cold biome should be receiving more attention. The temperature sensitivity of soil respiration is a key parameter in the estimate of C budget, and a comprehensive understanding of how the impacts of N addition and season variation on temperature sensitivity of soil respiration still remains need^11^,^12^.

Cold temperate forests in northeastern China experience seasonal snow cover, where snowpack is continuous for half of a year^17^. But most measurements of *R*s in the cold temperate forests of China were conducted during the growing season due to the difficulty in field measuring CO_2_ efflux in winter. However, in these cold biomes, seasonal snow cover is one of the most important variables controlling processes of forest ecosystem C cycle^18^. Changes in durations and depth of snowpack would lead to large differences in soil temperature, moisture, accompanied changed microbial activities^19^ and root activities^20^,^21^. Snow cover provides an insulated layer, providing a stable environment for cold resistant microorganisms^22^ and the continual microbial activities might lead to winter soil respiration^17^. The measurements of *R*s throughout an entire year to elucidate the seasonality of *R*s and its potential driving factors in this forest ecosystems is needed.

The main objectives of this study were to examine effects of enhanced N deposition on soil respiration during an entire year (both the growing season and winter). In brief, we want to (1) explore relationships of *R*s against soil temperature and moisture in different N addition treatments during the growing season and winter; (2) examine the effects of seasonal snow cover on winter *R*s; and (3) quantify annual and seasonal *R*_S_ in an entire year. We hypothesized that low-levels of N addition promoted *Rs* and high-levels of N addition inhibited *Rs*, and *Rs* was significantly decreased with the reduction of snowpack depth.

## Materials and Methods

### Site description

The experiment was conducted at Fenglin Natural Reserve of Leseer Khingan Mountains in Heilongjiang Province, Northeast China (48°02′–48°12′ N, 128°58′–129°15′ E). The reserve is a cold temperate forest characterized by a continental monsoon climate. Mean annual air temperature is −0.5 °C (1959–2013), with monthly mean air temperature ranging from −24.2 °C in January to 20.3 °C in July. Annual precipitation ranges from 680 to 750 mm. The snowpack lasts for 149 days in the measurement year. The forest is dominated by old-growth (210 years) spruce (*Picea koraiensis*), fir (*Abies nephrolepis*) and Korean pine (*Pinus koraiensi*) with a mean stand density of 972 ± 96 trees ha^−1^, a mean diameter at breast height (1.3 m) of 13.7 ± 7.5 cm and a mean tree height of 16.7 ± 5.3 m.

### Experimental treatment

The experiment was conducted within three random blocks, each consisting of four plots measuring 20 m × 20 m, and the plots were separated by 10-m wide buffer strips. Four N addition treatments were included in this study: control (no added N), low-N (5 g N.m^−2^.yr^−1^), medium-N (10 g N.m^−2^.yr^−1^) and high-N (15 g N.m^−2^.yr^−1^), with three replicates randomly distributed at each treatment^23^,^24^,^25^,^26^. Dilute ammonium nitrate (NH_4_NO_3_) solution was applied to the forest floor every half a month during the growing season (May to October) from 15^th^ May 2010. In each plot, fertilizer was mixed with 32 L of water (equal to 0.08 mm annual precipitation), and applied using a backpack sprayer below the canopy. Two passes were made across each plot to ensure an even distribution of fertilizer. The control plots received 32 L water without N addition. During the winter, we undertook five treatments of snow manipulation at each plot, including depth of 0 cm (no snowpack), 10 cm, 20 cm and 30 cm and natural depth. The natural snow depth in early winter, deep winter and late winter are 33 ± 5 cm, 42 ± 4 cm and 37 ± 5 cm, respectively. It should be noted that since the natural snow depth in early winter (33 ± 5 cm) is very close to the 30 cm snow manipulation treatment, for this period, we only took four snow manipulation treatments, including 0 cm, 10 cm, 20 cm and natural depth. 48 snow fences (1.6 m tall and 20 m long; 4 fences at each plot) were installed in 8^th^ October 2013, and once the depth of snowpack exceeded the prescribed snow depth mentioned above, a nylon mesh was fixed on the top of the fences to prevent further snow accumulation.

### Soil respiration and related environmental factors

Growing season *R*s was measured every two weeks from May 2013 to October 2013 and the winter *R*s was measured every two months during December 2013 to March 2014 (early winter, deep winter and late winter, respectively) considering the severe difficulties of the fieldwork in snow-covered conditions. Practically, for the early and the deep winter, measurements were carried out three days after a heavy snowfall event and for late winter we took the measurement before snow melting. Each observation lasted about a week. For each of 12 plots, we randomly inserted 3 polyvinyl Chloride (PVC) collars (20 cm inside diameter and 12 cm in height) for the growing season measurements and 42 collars for winter measurements (3 replicate collars for each snow manipulation treatment). The soil collars were inserted 9 cm into the soil and 3 cm left above the soil surface, one week before the N addition treatment in 2010. All measurements were taken with a Li-8100 Automated Soil CO_2_ Flux System (Li-Cor Inc, Lincoln, NE, USA) between 8:00–11:00 a.m. Each measurement was repeated 3 times for each collar to produce a collar’s mean *R*s rate. We calculated *R*_***S***_ using exponential regression model with the LI-8100 file viewer application software (LI-8100/8150 Instruction Manual). During the winter, the snow in the collars was carefully removed before measurements and *Rs* was recorded once the *Rs* had stabilized to avoid disturbance of remove snowpack. The Li-8100 was kept in an isolated and heated container to protect them from freezing and ensure normal operation.

**Figure 1:**
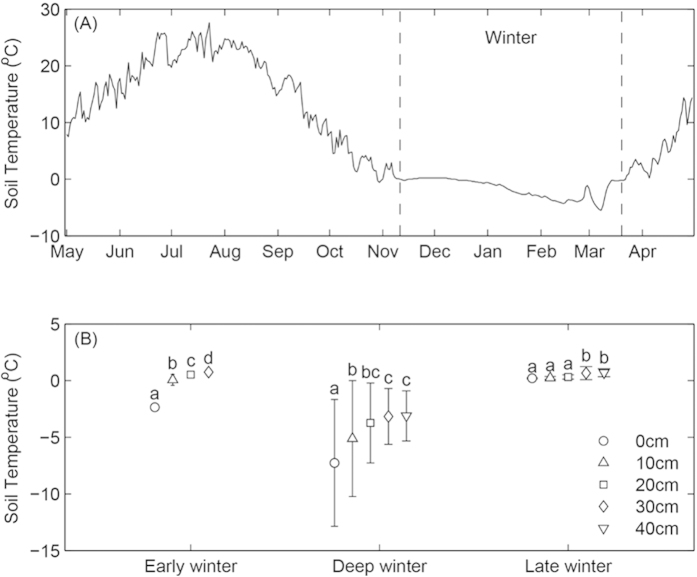
Variations of surface soil temperature (5 cm, A) and mean soil temperature (means ± sd) across different snowpack depth manipulations during different stages of the winter (B).

Discrete soil temperature at 5 cm below surface were monitored simultaneously with the measurement of *R*s, using a soil temperature probe (Omega Engineering Inc. USA) connected to Li-8100. Continuous soil temperature at 5 cm below surface soil was monitored hourly by Em-50 data logger (Decagon Devices, Inc. USA; Fig. 1A). Soil samples (O layer and the 0–10 cm soil layer) were taken at 5 randomly distributed points using a handheld auger in each plot in May and September of 2013 and May of 2014. The five soil samples at same soil layer were pooled to one sample. The soil samples were sieved (2 mm) to remove stones and roots, and then stored at −10 °C before analysis. The air-dried sub-samples of the same layer were mixed and ground, and filtered with 1 mm sieves for soil total C and total N analysis using an automated TOC/TN analyzer (multi N/C3100, Analytikjene AG, Germany). The soil pH measurements with air-dried soil were conducted in a 1:2.5 soil: water (distilled water) suspension using ACG 808 (Schott Gerate, Germany) digital pH-meter with glass combination electrode. Soil sub-samples (fresh soil) were extracted with 0.2 mol/L KCl solution, and the ammonium and nitrate contents in extracts were measured using indophenol method (spectrophotometrically at 655 nm) and uv-spectrophotometry method (spectrophotometrically at 410 nm) using a UV/VIS Aqumat Spectrophotometer (Thermo Electron Spectroscopy LTD, Cambridge, UK). Soil moisture contents were measured by oven-drying for 24 hours at 105 °C. The ammonium and nitrate were summed as the total inorganic N in the soil layers.

### Statistical analysis

The apparent dependence of *R*s on temperature was calculated according to the following equation:


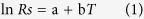


where ln *R*s is the natural log of soil respiration and *T* is the examined soil temperature at 5 cm below surface. The regression coefficients, a and b, were used to achieve the apparent temperature sensitivity (*Q*_10_) and the reference respiration rate (*R*_0_, approximates to *R*s at 0 °C) as follows:


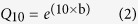



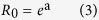


Both the *Q*_10_ and *R*_0_ were separately calculated for winter and the growing season at each N addition treatment. The difference of *Q*_10_ (or *R*_0_) was statistically tested using analysis of covariance (ANCOVA).

To assess the contribution of winter *R*s to that of the whole year, we constructed *R*s-T models. Compared to the other models (i.e., *Q*_10_ model (Eqn. 1), Michaelis-Menten kinetics model^27^ and Lloyd-Taylor model^2^), the Gamma model proposed by Khomik *et al.* (2009)^28^ performed better in simulating the *Rs*-T relationship and could be expanded to help analyze *Rs*-T relationship in the context of other environmental factors, such as soil nutrients. We used Gamma model to assess the contribution of winter *R*s to that of the whole year at the different quantities of N additions aim to test whether N addition had a significantly impact on contribution of winter *Rs*.

Gamma model was expressed as following:


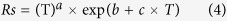


where T is (T_soil_ + 40), a, b and c are regression coefficients. T_soil_ is soil temperature under 5 cm below surface.

For each N addition treatment, we separately developed *R*s-T models for winter and the growing season (in winter only *R*s under natural snow cover was used) in each replicate plot. Then we applied the models to the continuous daily average soil temperature data to calculate the modelled time series of *R*s. The start of winter (or the growing season) is defined as the first day when 7-days running mean soil temperature <0 °C (or ≥0 °C) for at least five consecutive days (Fig. 1A). Two-way ANOVAS were used to examine the effects of N addition and snowpack depth treatments and their interactions on the soil respiration during different stages of winter. One-way ANOVA with Tukey’s HSD test was used to examine the differences in temperature sensitivity (Q_10_) and the reference respiration rate (R_0_) during the winter and the growing season. All statistical analyses were performed in using R package (v.2.15.1).

## Results

### Effects of snowpack and simulated N addition on winter soil respiration

**Figure 2:**
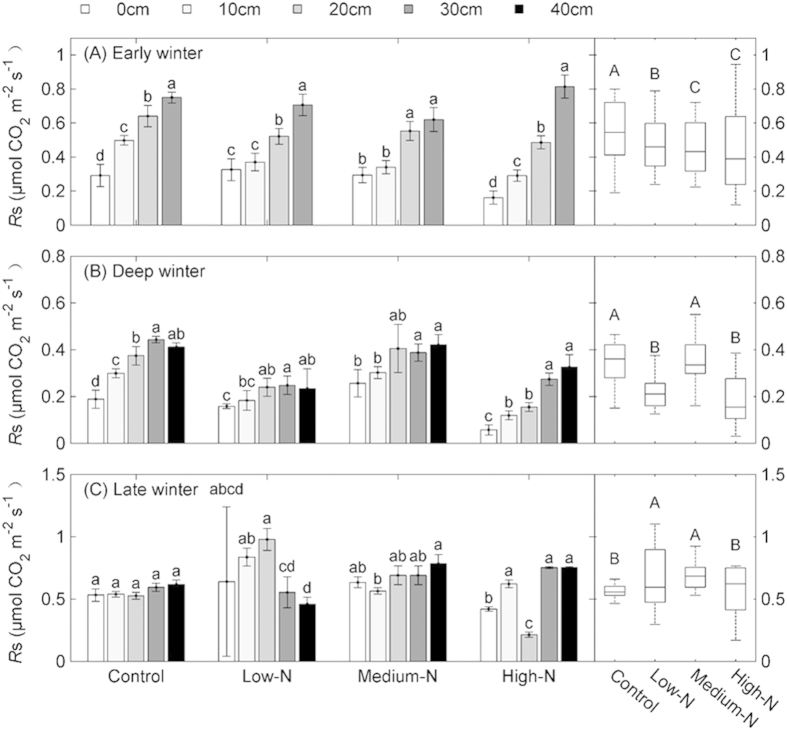
Soil respiration (*R*s) at four quantities of nitrogen addition (control, low-N, medium-N and high-N) treatments and different snowpack depth manipulations during early winter (A), deep winter (B) and late winter (C) (Left); and *R*s at different quantities of nitrogen additions treatments (Right). Different letters denote significant differences at *P* < 0.05. Data are means ± sd.

The effects of different depths of snowpack on soil CO_2_ efflux varied across different periods of the winter (Fig. 2). Soil CO_2_ efflux was significantly decreased with the reduction of snowpack depth during both early and deep winter (F = 692.53 and 169.04, respectively; *P* < 0.001; Table 1). Across four quantities of N addition treatment, *R*s increased from 0.18–0.37 μmol CO_2_ m^–2^ s^–1^ for the snowpack removed treatment to 0.69–0.82 μmol CO_2_ m^–2^ s^–1^ for the natural snowpack (Fig. 2A) during early winter and from 0.13–0.27 to 0.37–0.45 μmol CO_2_ m^–2^ s^–1^ for deep winter (Fig. 2B). No statistically significant increases of CO_2_ efflux were detected along a gradient of snowpack depth during the late winter (Fig. 2C).

In addition, the effects of simulated N addition on soil CO_2_ efflux treatments showed different patterns among the different periods during the winter. For the early winter, the soil CO_2_ efflux at low-N, medium-N and high-N treatments were significantly decreased by 12%, 17% and 20% compared to that at control treatment (*P* < 0.001), respectively. Similarly, compared to control during the deep winter, the CO_2_ efflux at low-N and high-N treatments decreased by 38% and 46%, respectively (*P* < 0.001), but was not significantly different for medium-N. For the late winter, the soil CO_2_ efflux peaked at low-N and medium-N treatment (24% and 20% higher than that of control (*P* < 0.05), and not significantly at high-N treatment. Overall the winter *R*s did not differ significantly at the different quantities of N additions treatments under natural snow fall (Table 2).

### Effects of simulated N addition on the growing season soil respiration

**Figure 3:**
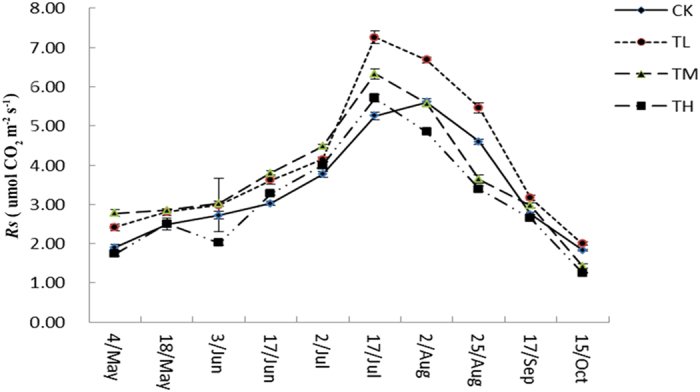
The different quantities effects of simulated nitrogen addition (CK, control; TL, low-N; TM, medium-N; and TH, high-N) treatments on the growing season soil respiration.

Overall *Rs* in the growing season showed a significant seasonality with the minimum occurring in October and the maximum occurring in late July (except the control) (Fig. 3). Soil CO_2_ efflux in the growing season ranged from 1.83 to 5.60, 2.00 to 7.26, 1.43 to 6.33 and 1.26 to 5.71 umol CO_2_ m^−2^ s^−1^ for control, low-N, medium-N and high-N treatment, respectively. Significant increase the rates of *Rs* in the low-N treatment in the growing season (Fig. 3). The medium-N treatment values measured in early August were higher than those of the controls, but the trend to reverse in late August (except September) (Fig. 3). The increase in soil CO_2_ efflux caused by high-N treatment was only found in late June and July during the growing season (Fig. 3). However, we found that low-levels of N addition significantly promoted *Rs*, contrary to the high-levels of N addition.

### Effects of simulated N addition on temperature sensitivity of soil respiration

**Figure 4:**
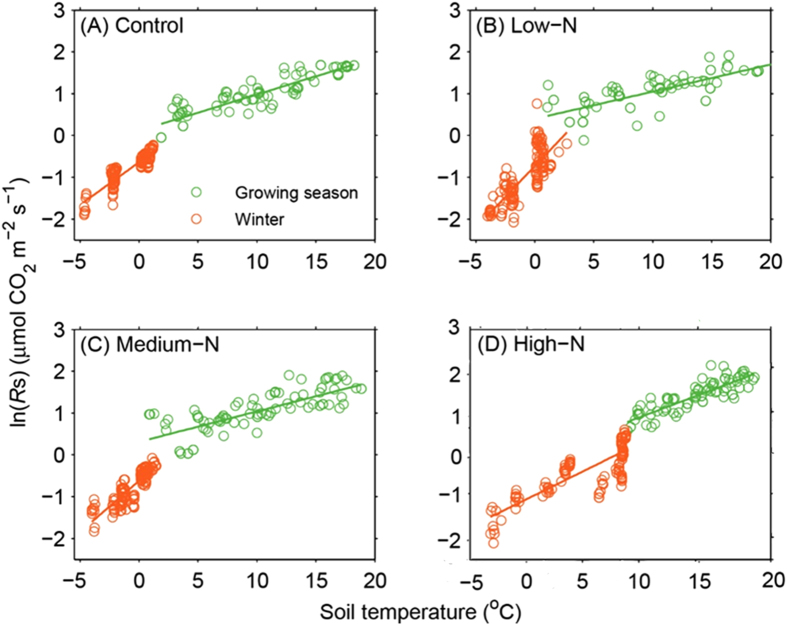
Differences of *R*s temperature dependence between the winter (orange) and the growing season (green) at the different quantities of nitrogen additions (A, control; B, low-N; C, medium-N; and D, high-N) treatments.

The exponential regression models showed that the temperature sensitivities (*Q*_10_) of the winter soil respiration were significantly higher than those of the growing season (*P* < 0.01; Fig. 4). The *Q*_10_ values of the winter were 7.7, 19.1, 11.6 and 3.1 for control, low-N, medium-N and high-N treatment, respectively, which were higher than the corresponding *Q*_10_ of 2.4, 1.9, 2.1 and 2.4 for the growing season, respectively (*P* < 0.01; Table 3). Within winter, the *Q*_10_ values of low- and medium- N treatments were higher than that of control, but much lower *Q*_10_ value was detected under the high N addition treatment than that of control (*P* < 0.01; Table 3). For the growing season, the *Q*_10_ at low-N treatment was significantly lower than that of control, but not significant at medium- N and high- N treatments (Table 3).

### The contribution of winter *R*s to annual *R*s

The predicted annual *R*s was 974.3 ± 67.1 g C m^−2^ yr^−1^ (the values of Rs-T model for winter and the growing season) in this cold temperate conifer forest without N addition treatment and winter *R*s (the start of winter is defined as the first day when 7-days running mean soil temperature <0 °C for at least five consecutive days) (46.8 ± 1.2 g C m^−2^) comprised 4.8 ± 0.3% of the annual total. Low and medium quantities of N addition exerted negative effects on winter *R*s and decreased it by 24% (low-N) and 10% (medium-N) compared with the control. High-N increased the modeled winter *R*s to 62.4 ± 1.7 g C m^−2^. Similarly, the contribution of winter *R*s to annual total was declined under low-N and medium-N treatments (3.6 ± 0.6% and 4.3 ± 0.4%, respectively) and increased under high-N (6.4 ± 0.5%).

## Discussion

### Seasonal snow cover changes winter *R*s

Several studies stated that seasonal snow cover creates an abiotic environment that is more insulated, stable and favorable for soil respiration due to relatively higher soil temperature^17^,^29^. Our study also observed the positive effects of snowpack on soil temperature (Fig. 1B) and *R*s (Fig. 2). On the other hand, decrease in snowpack depth will induce more soil freezing and thawing events, we observed that *R*s was suppressed by a reduction in snowpack depth during the early and deep winter (Fig. 2A,B), which suggested that the effect of insulation was dominant in this periods of winter. However, during the late winter, when soil experiences considerable numbers of freezing and thawing cycles^17^,^30^,^31^, the positive effects of snow insulation on *R*s was largely offset by decline in freezing and thawing cycles. As a result, a weak relationship between *R*s and snowpack depth was detected during the late winter in this study (Fig. 2C). Results of suppressed soil respiration after the artificial decreasing snowpack depth suggest a potential positive feedback to climate change which may induce thinning snowpack in the future^32^.

### Effect of N addition on *R*s and its temperature sensitivity

Our results demonstrated that artificial N addition significantly suppressed *R*s during the early and deep winter. Winter soil respiration is predominantly comprised of heterotrophic respiration that is mainly controlled by microbial community. Previous studies in temperate forests demonstrated that N addition decreased soil microbial biomass^33^,^34^, which may decrease the heterotrophic respiration from microbial community in growing season. This suppressed effect of N addition on soil respiration occurred during the winter in this study, which suggested that N addition changed the activity of cold-tolerance microorganisms^35^. In addition, Tucker (2014)^20^ found root respiration in mountain forests during winter, and N addition may inhibit root respiration to decrease of Rs during winter.

The *Q*_10_ of *R*s in this study fluctuated from 1.9 to 2.4 along a N addition gradient for the growing season and from 3.1 to 19.1 for the winter, compatible to the range of this parameter in previous studies^36^,^37^. Besides, the *Q*_10_ values during winter are significantly higher than those of the growing season through all N addition treatments in our analysis. High *Q*_10_ values at temperatures below 0 °C were also reported by a number of estimations under both laboratory conditions^38^ and field experiments^17^. Several mechanisms have been introduced to explain this difference in *Q*_10_, including changes in substrate supply and in soil microbial community at the cold circumstances^39^,^40^. Recently, Tucker (2014)^37^ explained this marked increase in *Q*_10_ below 0 °C as a result of reduction in unfrozen water for substrate diffusion following the conversion of liquid water to ice. The content of unfrozen water in soil shows an exponential relationship between soil temperatures below 0 °C^41^, adding an additional source of temperature sensitivity to *R*s. Likewise, Lipson *et al.* (2009)^42^ pointed out that seasonal variation in Q_10_ was linked to changes in the composition of the microbial community, and soil microbial communities from under-snow had higher Q_10_ values than the summer and fall communities.

N addition also affected the magnitude of *Q*_10_; however, the effect exhibited a significant variation between the growing season and the winter. During the winter, a low quantity of N addition significantly increased *Q*_10_ from 7.7 to 19.8 while the high N addition decreased the magnitude to 3.1. During the growing season, the variation in *Q*_10_ between different N addition treatments became marginal and statistically non-significant except for a slight decrease in *Q*_10_ under the low N addition treatment. This inconsistency in the response of *Q*_10_ to N addition might reflect the distinct sensitivity of microbial respiration and root respiration to N. Since the winter *R*s is predominantly composed of microbial respiration which is more susceptible to availability of N than root respiration, the *R*s during the winter is likely to be affected by the manipulation in soil N^43^. Furthermore, the different direction of the *Q*_10_ response to low and high N addition treatments during the winter might suggest a potential change in metabolic pathways^44^ or microbial composition^19^ in soil; however, the specific explanations for different response of *Q*_10_ to different quantity of N addition between seasons still remain unclear.

### The contribution of winter *R*s to annual *R*s along a N addition gradient

In the present study, we modelled the cumulative winter *R*s as 46.8 ± 1.2 g C m^−2^ and the contribution of winter *R*s to annual total as only 4.8 ± 0.3% for the control plot. Our estimates of winter *R*s and its contribution are both within the range of previously reported ones for temperate forests (21.6–84.3 g C m^−2^ and 3–15%)^17^,^45^,^46^,^47^,^48^,^49^ and boreal forests (55.0–139.1 g C m^−2^ and 8–25%)^50^,^51^. In cold temperate forests of China, the previous estimates of winter *R*s ranged from 22 to 53 g C m^−2^ 17,49, most of which are lower than that of ours. The CO_2_ efflux through soil respiration is not a constant through space and time even for a specific vegetation type and is rather subject to differences in definition of winter^17^, occurrence of extreme event^52^, substrate availability^40^, disturbance, freezing and thawing events^53^, snow insulation^39^, measurement technique^46^ and statistical analyses.

Variation in estimated cumulative winter *R*s among similar forest ecosystems is partly due to the differences in winter snowpack pattern and accompanied soil temperature^17^. The depth of natural snowpack (33–42 cm through snowpack duration) of our observation was higher than the one (<30 cm) of Wang *et al.* (2010)^49^ and another (around 30 cm) of Wang *et al.* (2013)^17^. Deep snow cover, which acts as an insulating layer, provides a stable respiration soil condition accompanied higher soil temperature around 0 °C^22^.

We also observed that N addition considerably influenced the contribution of winter *R*s to annual total. However, the direction of this influence differed between the low and the high quantity of N addition. Specifically, low N addition suppressed the proportion of winter *R*s to annual *R*s while high N addition increased it. These distinct responses between different N treatments might partly due to the lower *Q*_10_ value for high N addition than that of low N treatment (Table 3), which limited the decrease in *R*s under extremely cold temperature. Moreover, a low and medium quantities of N addition also increased the growing season *R*s (Fig. 2), thus the proportion of winter *R*s further declined.

## Conclusions

Snowpack enhanced winter *R*s mainly through snow-depth-dependent insulation of lower soil temperatures, however, effect of N addition was observed in our study. The season variability of *R*_*S*_ is crucial for estimating global carbon cycle and atmospheric CO_2_ concentration. Estimated Q_10_ values under N addition were heterogeneous in temporal pattern (season variability). Under all N addition treatments, *Q*_10_ of winter *R*s was significantly higher than that of growing season *R*s. Low-N and medium-N addition significantly increased *Q*_10_ of winter *R*s, but high-N addition decreased it. Winter *R*s contributed minor to the annual *R*s (ranged from 3.6% to 6.4%) in the cold temperate conifer forest of China, but given the sensitivity of winter *R*s to the snowpack depth and nutrient availability, the ongoing climate change may have the potential to alter the annual carbon flux of cold temperate conifer forest in China. It is very important to understand the effects of N addition and temporal pattern on *Rs* to accurately predict soil CO_2_ flux in forest ecosystems under a changing climate.

## Additional Information

**How to cite this article**: Liu, B. *et al.* Annual soil CO_2_ efflux in a cold temperate forest in northeastern China: effects of winter snowpack and artificial nitrogen deposition. *Sci. Rep.* **6**, 18957; doi: 10.1038/srep18957 (2016).

## Figures and Tables

**Table 1:** Results of the two-way ANOVA for the effects of nitrogen addition and snowpack depth treatments and their interactions on the soil respiration during different stages of winter.

	Early Winter			Deep Winter			Late Winter		
Sources of deviation	df	*F*	*P*	df	*F*	*P*	df	*F*	*P*
N	3	39.23	<0.0001	3	265.83	<0.0001	3	20.878	<0.0001
Snowpack	3	692.53	<0.0001	4	169.04	<0.0001	4	4.711	<0.01
Interaction	9	21.92	<0.0001	12	12.17	<0.0001	12	25.45	<0.0001

Abbreviation: N, nitrogen addition.

**Table 2:** Selected soil characteristics and winter soil CO_2_ flux at the different quantities of nitrogen additions treatments under natural snow fall.

	Control-N	Low-N	Medium-N	High-N
Winter Soil Temperature (°C)	−0.19 ± 1.52	−0.21 ± 1.24	0.14 ± 1.13	−1.96 ± 3.50
Winter Soil Moisutre (%)	0.54 ± 0.34	0.63 ± 0.45	0.64 ± 0.44	0.60 ± 0.46
pH	4.58 ± 0.34	4.58 ± 0.34	4.60 ± 0.35	4.60 ± 0.35
Soil Carbon Density (kg C·m^−2^)	14.28 ± 5.87	14.41 ± 4.67	14.83 ± 5.69	14.85 ± 3.94
Soil Nitrogen Density (kg N·m^−2^)	2.03 ± 0.59	1.96 ± 0.64	2.01 ± 0.72	2.15 ± 0.52
Winter Soil CO_2_ Flux (μmol CO_2_ ·m^–2^ ·s^–1^)	0.59 ± 0.15	0.47 ± 0.21	0.61 ± 0.17	0.63 ± 0.23

The differences of all parameters are not statistically significant.

**Table 3:** Temperature sensitivity (*Q*_10_), the reference respiration rate (*R*_0_) and regression models coefficients during the winter and the growing season.

Specified periods		Control	Low-N	Medium-N	High-N
Growing Season	*Q*_10_	2.4 Aa	1.9 Abc	2.1 Aac	2.4 Aad
	*R*_0_	1.12 Ab	1.49 Aa	1.37 Aa	1.11 Ab
	a	−0.15	0.16	−0.21	−0.25
	b	0.16	0.08	0.20	0.06
	c	0.10	0.06	0.11	0.11
Winter	*Q*_10_	7.7 Ba	19.1 Bb	11.6 Bab	3.1 Bc
	*R*_0_	0.53 Ba	0.48 Ba	0.54 Ba	0.47 Ba
	a	1.99	−0.50	−5.97	−6.61
	b	3.84	−1.05	−9.75	−17.98
	c	−4.02	0.46	10.61	34.25

Regression models of soil CO_2_ efflux against soil temperature at the 5 cm depth for the specified periods. The regression models are of the form: *Rs* = (*T*)^*a*^ × *exp*(*b* + *c* × *T*), where a, b and c are regression coefficients.

Abbreviation: Letters within a column represent significant differences of *Q*_10_ and *R*_0_ between the growing season and winter (capital letters) and across different quantities of nitrogen addition (lower cases) at *P* < 0.05.
